# Indents

**Published:** 1914-12-15

**Authors:** 


### INDENTS.

☞Brief Articles of Technical or Scientific Value will receive notice and comment. Contributions invited.

*Cavity Preparation.* Much discussion as to the form of cavity for successful inlay work has taken place in the past five years. For gold fillings made by the malleting methods anchorage of some form is essential because mechanical retention is necessary. For the cemented inlay mechanical retention is not always required. Conzet says that the only difference between a proper preparation for a gold filling and a gold inlay, for a proximo-occlusal cavity, is that for the former at least a slight undercutting is necessary, while for the latter no undercut is admissible. Some operators in porcelain inlay work seek for mechanical form of a decidedly retentive character, while others disregard entirely retentive form and even deprecate it.

*Decay of Children's Teeth.* Edward F. Brown, in the *Journal of Allied Societies*, says that if a procession of all the children in New York City who had decayed teeth were to march single file past any given point, it would take nearly a month of constant day and night marching to review the parade. That is something to think about.

*Germany's Experience with School Dentistry.* After many years' experience in caring for school children's teeth, it has been found that the effort is worth while; because, less time is lost from school attendance than was lost from toothaches before the dental work was made compulsory; the cost of the dental care was not so much as was formerly expended on hospital expenses; and finally because of better health, because the teeth are cared for, the child made better progress in his studies and he was less irritable and easier to control.

*Navy Dental Surgeons.* Out of 65 naval dental surgeons 55 have joined the National Dental Surgeons as component members. The other ten are in the far distant countries and they will also join as soon as they can be made acquainted with the advantages of the Association.

*International Congress Delegates.* By a resolution of the House of Representatives of the nation, the invitation of King George, of England, was accepted and 15 official delegates were nominated to represent this country in the Sixth International Dental Congress which met in London, England, August 3rd.

*Dental Educaion of the People.* The House of Delegates of the National Association approved the report that a committee on Dental Hygiene and Public Education be authorized to prepare a book on dental hygiene for public instruction. Such a book, if properly edited, would be of great service to schools desiring to introduce this instruction.

*Iodoform Deodorizer.* The odor of iodoform can be removed from the hands by washing the hands and placing a small quantity of pulverized mustard in the palms while wet and rubbing thoroughly over every part of the hands and then washing them with soap and water. Instruments can be deodorized by allowing them to stand in mustard paste for an hour or so.—*Medical Bulletin.*

*Nitric Acid as a Solvent of Vulcanite.* Dr. J. L. Kaufman in the *Cosmos* says that the remaining vulcanite which sticks to porcelain teeth when removing them from an old plate, may be easily detached by placing the teeth in a test tube of nitric acid. The acid may be heated over a bunsen flame, and the process will be much accelerated.

*Grading its Dental Servants.* The Standardization Committee of the New York City Board of Estimates will grade its dental employees into three groups. The first group will be the attendants who assist in operations with salaries of from $240 to $360 for eighteen hours work each week. The second group will contain dental graduates or licensed dentists who perform the usual operations of filling and treating teeth, with salaries of from $900 to $1,380 per annum for 18 hours work each week. The third group will contain dentists of more experience and exceptional skill, who shall supervise the work of the two lower groups; they must have had two years of work in group two class or its equivalent. The salary in this group will be $1,560 and up depending on length of service and experience. As in the other groups at least eighteen hours of service each week is demanded. Promotion from one group to another is based on experience and capacity. This is, we suppose, necessary in the conduct of such affairs in a large city and perhaps it is all right, but it seems unnecessary to put a professional man into such restraints. Perhaps it is necessarily a part of the machine methods of doing things in large cities. We trust it may not become such a political organization that its efficiency will be impaired.

*Pyorrhea Technic.* Always have at hand generous quantities of a warm aseptic mouth-wash, and I want to insist on its being warm all the time, as I consider it a species of brutality to flush sensitive surfaces with cold water. I find the ordinary chipblower most useful for this purpose, as it furnishes a free and copious flow of liquid. An occasional use of warm spray, with thirty-five pounds pressure for forcing out debris, is also beneficial.

For asepsis I have a sterilizing tray conveniently placed, containing a five per cent solution of Lysol, into which instruments are dropped whenever they are to be changed. An assistant dries them and places them in proper place. A glass containing a small quantity of ten per cent solution Lysol is convenient, into which instruments are dipped previous to their introduction into the mouth. A basin of water is adjacent to the chair containing two per cent Lysol solution for frequent rinsing of hands. We are now ready for anesthesia, which is done simply by flowing a four per cent solution of cocaine or novocaine (with one drop of carbolic acid to the ounce to keep sterile) into the pockets, and around the cervical margin. Next, massage the medicine well into the tissue, and repeat as often as required. Don't use adrenalin in this solution, as free blood letting is beneficial; and don't inject, for fear of spreading infection. After anesthetizing the parts I select a suitable file-shaped scraper and remove all excrescences or deposits from all affected area. With this instrument I can get a perfectly smooth surface, but I still further endeavor to smooth the surface by using a sharp blade of suitable cutting shape, and cover the entire area again, by which work I get the roots into a suitable condition for the final finish or polish.

With razor edged cutting instruments I go over the entire surface for the third time, using long, light strokes, taking care that each stroke shall overlap the preceding one, until the surface has the smoothness of glass. Here again I caution you against using short strokes, as in this way it is very easy to cut shoulders into the surface, which are hard to obliterate without cutting too deep. After this finishing process is complete, I introduce lactic acid to the full depth of the pocket, apply iodine to the surfaces and the work is done. It is of the utmost importance that you reach the bottom of every pocket. The instrument should rest solidly against the bone, and begin your operation at the junction of tooth and bone, working backward from the point of the instrument, when using a curved blade, to prevent injury to soft tissues.

You will frequently find irregular surfaces of bone, due to unequal absorption, and in such cases the bone must be curetted to a smooth surface also.—Dr. Julian Smith, in *Western Journal*.

*Arsenic Pentoxid.* The therapeutics of arsenic for dentists is the simple one of devitalizing pulp tissue, and the application of arsenic pentoxid is essentially the same as that of the trioxid, except that you may—I would advise it—double the dosage, since the pental arsenic is about one-half as toxic as the triad. With regard to the arsenate, particularly sodium arsenate, which is extremely soluble, I would advise you to employ a saturated solution. A pledget of cotton soaked with this solution may be applied under a cement filling, which must be inserted without pressure; or the gutta-percha arsenic cup, which can be had at the dental depots, may be advantageously used in this connection. Of course, the sodium arsenate may be used in paste form like the oxid. The best vehicle for arsenic pentoxid is glycerin, as every other paste would deteriorate from the oxidizing property of this arsenic. Add the powder to a small quantity of glycerin until a suitable paste is obtained; should the preparation become rather fluid, add powder from time to time.

About three years ago I conceived the idea of testing the pentad arsenic in the devitalization of the pulp, and asked my druggist to procure a sample of the drug. As he could not obtain it in Cincinnati, I prepared it for myself. An aching tooth in my own mouth at the time needed to be treated, and I asked Dr. Siegel to apply this preparation in my case. From former experience of pain I had suffered from the trioxid I considered myself in a fair position to judge of the result. We allowed the treatment to remain in the tooth one week. I assure you I felt no effects—indeed, I supposed no action had taken place; on opening the tooth, however, we found the pulp entirely devitalized. After this I applied arsenic pentoxid in my own practice with very pleasing results. No pain was ever reported. I found I could apply it on highly inflamed pulps, even relieving my patients of their suffering before they left the chair. Another advantage I found is that one can allow the arsenic to remain in a tooth indefinitely without bad consequences. I applied it in a patient's tooth and removed it again after her sojourn in Europe for three months. I remember two cases—one an upper first molar, another a lower first molar,—where I applied the pentoxid three years ago, and the treatment has not yet been removed, the teeth having given no trouble in the interim.—Dr. Geo. F. Fette, in November *Cosmos*.

*Safe and Easy Method of Applying Silver Nitrate to Teeth.* In applying silver nitrate to teeth there is danger of particles coming in contact with the soft tissues, or of being swallowed, both of which accidents may be of serious consequence. A suitable wire point of Victoria metal is heated over a flame, and dipped into a bottle in which the silver nitrate crystals are being kept. The crystals will adhere to the hot wire, and can be melted to form a point or bead, which is applied to the desired portion of the tooth. If the silver is to be applied in the interdental space, the drug is melted in a similar manner to a thin bronze wire as employed in orthodontia. After use the wire point is again held in a flame and the silver nitrate is burned off, leaving a coating of pure silver. In this way accidental contact with the operators' fingers, linen, etc., is avoided.—F. Zader, *Deutsche Zahnaerztliche Wochenschrift.*

*Matching Teeth in the Mouth.* When matching the shade of a natural tooth as a guide to the selection of teeth for a denture, a large mouth-mirror should be placed behind the specimen artifical tooth when in position beside the natural one. This will show up the two teeth and allow of a very much better comparison.—*Edwards' Dental Quarterly.*

*Protecting the Pulp Under Silicate Cement Fillings.* A simple means for protecting the pulp under a silicate cement filling consists of a pellet of white gutta-percha of suitable size. White gutta-percha is preferable to pink, as silicates are inserted chiefly in anterior teeth and the pink gutta-percha might shine through the filling.—*Zahnaerztliche Rundschau.*

*Filling for Absorption in Pyorrhea Treatment.* In the past year I have been experimenting somewhat in filling the spaces between the roots of the molars, where the bone has dissolved away, with satisfactory results for this period. After polishing these surfaces as described with a sharp round bur I go over the surface of the bone and root and round out the angles. Then use twenty-five per cent trichloracetic acid to cauterize and control hemorrhage and dry out; then flow copper cement from a Jiffy tube and fill the spaces, using the fingers on both sides of the tooth to conform the cement to the loose gum tissue. Whether this will prove an irritant or not time will tell, but up to the present time only beneficial results have been noted.—Dr. Smith in *Western Journal.*

*Systemic Infection from the Mouth.* I had a patient referred to me two weeks ago with rheumatism. Her left hand was so swollen that she could not close the fingers. Her physician said, "We have examined her tonsils, her ears, her nose and throat, her everything else that we could think of and we have not found a focus of infection. Now it is up to you to tell us whether or not her teeth are causing it." In other words, they have excluded everything else and the future health of that woman is being put up to the dentist. In the examination of this particular woman's mouth I have found pus present in pockets about three teeth, and there were pockets without pus about many others. I had radiographs made. These showed more or less absorption of the alveolar process about practically every tooth in both jaws. The woman is about 38 years of age; she has as beautiful a set of teeth as anybody could wish to see, has never had a tooth extracted, has seldom had one filled. Now, it is rather a serious matter to decide that that woman shall have all of those teeth extracted. I have not decided that yet, but I have decided to extract quite a few of them. It is a case that makes one stop and think seriously as to what our best duty is to such an individual. I was talking to an older practitioner a few days ago who said that since these matters had come up he had been checking over a list of patients that he had on his books who had come to him years ago with pyorrhea. He told me they were all dead, practically every one of them has died. Possibly statistics would show that people with pyorrhea die at an earlier age, on the average, than others, from an illness secondaiy to the mouth infection.—Dr. A. D. Black, in *Western Journal* for October.

*A Method of Treating Pyorrhea.* *First sitting*: Examine mouth and mark on examination sheet all hopelessly loose teeth, decayed or abscessed teeth, mal-occlusion, ill-fitting crowns and bridges. Agree on fee per *sitting*, "which sitting should never exceed one hour of time." Require a skiagraph to be obtained by the next sitting. Spray the gums with some mild antiseptic, using not over fifteen pounds pressure. Paint the gums with tincture of iodine. Dismiss the patient and charge her up with one sitting.

*Second sitting:* Spray mouth with normal salt solution and paint gums with tincture of iodine. Extract all hopelessly loose teeth, remove all ill-fitting crowns and bridges (and that means most of them), and clean out all cavities and fill with cement or amalgam. Should the skiograph disclose abscessed or necrossed condition, this should have at least, some attention at this time. Spray the mouth with some mild antiseptic and dismiss the patient.

*Third sitting:* Spray the mouth with normal salt solution, to which has been added some aromatic water, such as peppermint, cinnamon or wintergreen. We are now ready to begin scaling and polishing the teeth. Let us make "one tooth at a time" our motto, and that tooth should be scaled and polished to completion before beginning another. First paint the soft tissues around the tooth with tincture of iodin and then proceed with the scaling. Use great care not to lacerate the tissues more than possible. Use only sterile water to wash away the debris during the operation. If the work is done thoroughly, I find in a case of the second stage, about two teeth will require an entire sitting for the average operator. I usually begin with the lower centrals If they are loose, when I have finished all I can do in one operation, I ligate them in such a manner as to hold them steady. Do not flood the pockets with antiseptics. While working with them I use water or salt-solution to wash away the debris, but when I am done I want the pocket to fill up with nature's own dressing—blood clot. I never want to explore that cavity again. Let it have a chance to get well. I continue with the remaining teeth at subsequent sittings, until all have been cared for.

Remember to first flush out the mouth, or preferably spray with normal salt solution, then paint gums around teeth to be worked on with tincture of iodine. There should be no need of disturbing these teeth again. After all teeth are cared for as stated, if necessary, construct and set your retaining appliance and correct malocclusion with anatomically constructed fillings, crowns and bridges, constructed a'ong sanitary lines. At the very first sitting, and at every subsequent one, preach into the patient the gospel of oral hygiene. Prescribe a suitable antiseptic mouth wash and teach her how and when to clean her teeth. I am aware that this method may receive severe criticism. I am a general practitioner and cannot vouch for a scientific accuracy in all my work, but I do in most cases get very pleasing results and a compensation commensurate with the service rendered.—V. B. Newell, in *Dental Review.*

*Caution in Casting Aluminum.* Aluminum is considered a connecting link between the noble and base metals, nevertheless it is base and has the property of absorbing its oxides, also markedly, the property of occluding gases. This latter property is evidenced by the odor of garlic (carbon compound) often noticeable in a newly made casting, also the lack of uniform density shown by burnishing a buffed surface of the metal. This implies that aluminum that has once been melted in a dental laboratory should be discarded. Upon the second or third melting of the metal a yellowish tinge may be noticed, which indicates superficial oxidation. Should the color appear with the first melting of the metal, it would indicate that the metal has been overheated, and that the casting, to that extent, is defective.—Dr. Wilson in the *Summary*.

